# Treatment with the SGLT2 inhibitor luseogliflozin improves nonalcoholic steatohepatitis in a rodent model with diabetes mellitus

**DOI:** 10.1186/s13098-015-0102-8

**Published:** 2015-11-19

**Authors:** Shirong Qiang, Yusuke Nakatsu, Yasuyuki Seno, Midori Fujishiro, Hideyuki Sakoda, Akifumi Kushiyama, Keiichi Mori, Yasuka Matsunaga, Takeshi Yamamotoya, Hideaki Kamata, Tomoichiro Asano

**Affiliations:** Department of Medical Chemistry, Division of Molecular Medical Science, Graduate School of Biomedical Sciences, Hiroshima University, 1-2-3 Kasumi, Minami-ku, Hiroshima city, Hiroshima 734-8551 Japan; Department of Internal Medicine, Graduate School of Medicine, University of Tokyo, 7-3-1 Hongo, Bunkyo-ku, Tokyo, Japan; Division of Diabetes and Metabolism, Institute for Adult Disease, Asahi Life Foundation, 1-6-1 Marunouchi, Chiyoda-ku, Tokyo, Japan

**Keywords:** Diabetes mellitus, Nonalcoholic steatohepatitis, SGLT2 inhibitor, Luseogliflozin

## Abstract

**Background:**

Insulin resistance with elevated glucose is a risk factor for non-alcoholic steatohepatitis (NASH). We investigated the effects of the sodium glucose cotransporter 2 (SGLT2) inhibitor luseogliflozin on NASH development using a rodent model.

**Methods:**

Mice were treated with both nicotinamide and streptozotocin (NA/STZ) to reduce insulin secretory capacity, and then fed a high fat diet containing trans fatty acids (HFDT) for 8 weeks. The NA/STZ HFDT-fed mice were divided into two groups, either treated with luseogliflozin or untreated, during this period. The glucose elevations in the NA/STZ-treated and HFDT-fed mice were significantly improved by luseogliflozin administration. While HFDT feeding induced NASH development as shown by liver weight gain with lipid accumulation and increased serum alanine aminotransferase, these changes were all attenuated in the group treated with luseogliflozin. In addition, fibrotic change and increases in collagen deposition with upregulations of collagen1 and smooth muscle actin and inflammatory cytokine expressions observed in the HFDT-fed mouse livers were also normalized by luseogliflozin administration.

**Conclusions:**

Taken together, these results obtained in mice demonstrate the favorable effects of administering SGLT2 inhibitors, for the treatment of NASH associated with diabetes mellitus. We anticipate that these agents would be applicable to humans.

## Background

Recent advances in the development of anti-diabetic drugs have provided numerous therapeutic options for patients with Type 2 diabetes mellitus (T2DM) [1–4]. Among various anti-diabetic drugs, sodium glucose cotransporter 2 (SGLT2) inhibitors are unique in terms of their mechanism of action. These drugs increase urinary glucose excretion, thereby lowering the blood glucose concentration [1, 5–10]. A number of previous studies, using rodent models, demonstrated that several of these SGLT2 inhibitors can ameliorate fatty liver with significant body weight loss, and the weight reducing effects of various SGLT2 inhibitors have also been documented in humans [11].

Nevertheless, to our knowledge, only one study to date has investigated the effects of a SGLT2 inhibitor on non-alcoholic steatohepatitis (NASH) development. In that study, ipragliflozin, a SGLT2 inhibitor, failed to reverse inflammation and raised both the alanine aminotransferase (ALT) and the aspartate aminotransferase (AST) level, despite only a slight reduction in hepatic lipid accumulation, in methionine choline diet (MCD)-induced NASH model rats [5].

Herein, we prepared a rodent model suffering from both DM and NASH, and obtained evidence that luseogliflozin exerts a strong protective effect against the development of NASH induced by a high fat diet containing trans fatty acids (HFDT).

## Methods

### Animals, diets and luseogliflozin treatment

**Fig. 1:**
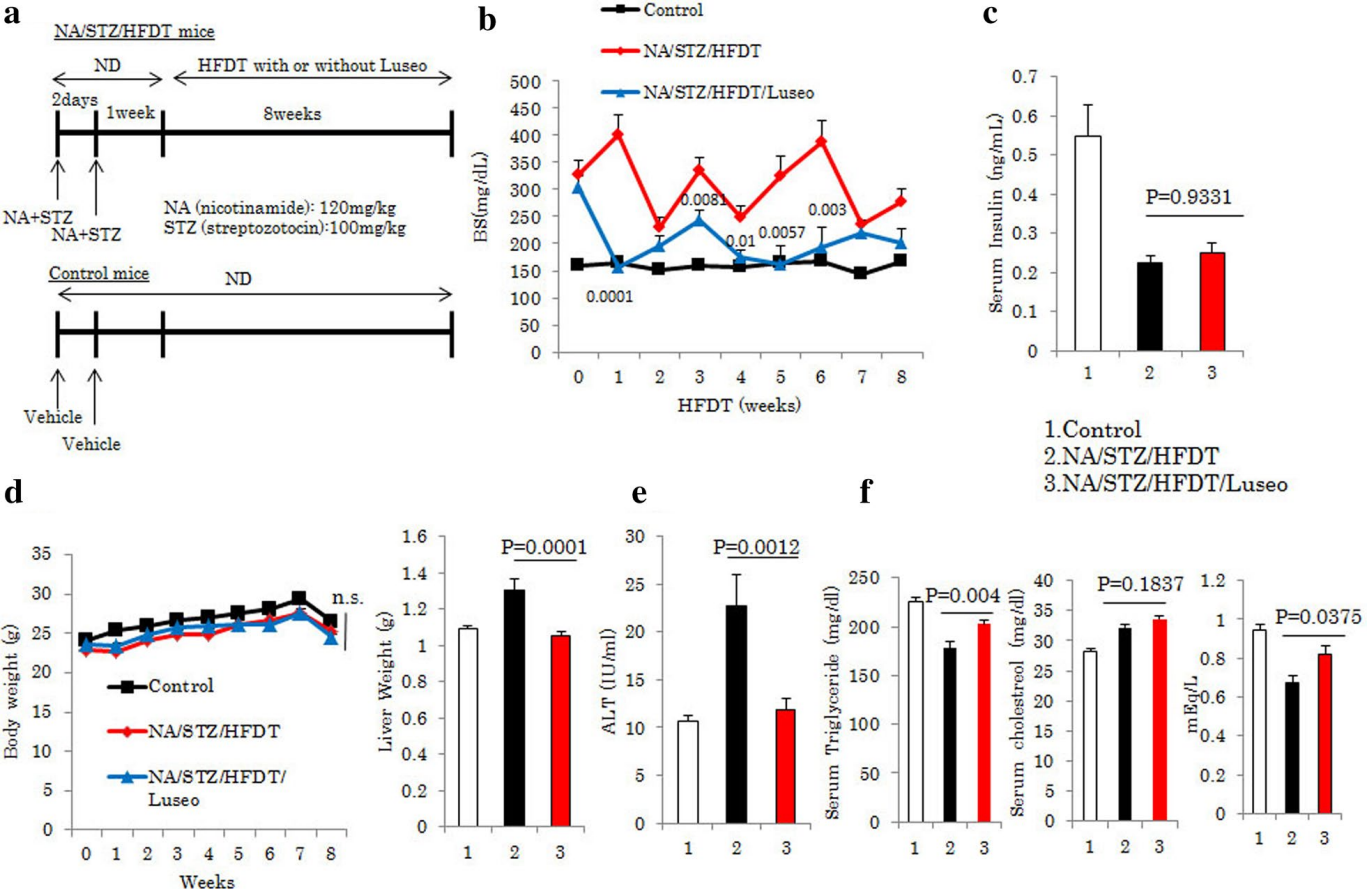
Luseogliflozin improved elevated glucose concentrations and normalized HFDT feeding-induced hepatosteatosis. **a** Control, NA/STZ/HFDT and NA/STZ/HFDT/Luseo mice. **b**, **c** Blood glucose and insulin concentrations in fasted states. **d** Whole body and liver weights. **e** Serum ALT level. **f** Serum triglyceride, cholesterol and non-esterified fatty acids (NEFA) levels. All data are shown as means + SEM

To induce mild to moderate diabetes in C57BL/6 mice, nicotinamide (NA) (120 mg/kg) and then streptozotocin (STZ) (100 mg/kg) was injected after starvation for 20 h, as shown in Fig. 1a. The mice were given a normal chow diet (ND) (Oriental Yeast, Tokyo, Japan) for 1 week as an acclimatization period, and then fed a ND or a diet with a high fat (40 % of kcal), high fructose (22 % by wt), and high cholesterol (2 % by wt) composition, wherein the fat source was trans-fat (Primex partially hydrogenated vegetable oil shortening, cat. no. D09100301, Research Diet, New Brunswick, USA). Luseogliflozin [TS-071: (1*S*)-1,5-anhydro-1-[5-(4-ethoxybenzyl)-2-methoxy-4-methylphenyl]-1-thio-d-glucitol], a SGLT2 inhibitor [12] synthesized by Taisho Pharmaceutical Co., Ltd. was given to half of the NA/STZ-treated and half of the HFDT-fed mice by mixing it into their food at a concentration of 0.1 %. This was done because the preliminary experiments suggested the maximal hypoglycemic effect to be obtained at 0.1 % (data not shown). As controls for this study, we used non-treated C57BL/6J mice fed the ND. All animals were handled in accordance with the Guidelines for the Care and Use of Experimental animals published by Hiroshima University.

### Histochemical studies

Paraffin-embedded liver sections were stained with hematoxylin and eosin for quantification of steatosis, fat droplets and inflammation of hepatic tissue. For detection of collagen deposition, deparaffinized sections were submerged in Sirius red solution.

For α-smooth muscle actin (SMA) staining, deparaffinized sections were permeabilized in 0.1 % Triton solution and heated in 10 mM citrate (pH 6.0). After being washed, the sections were incubated with SMA antibody (1:500) at 4 °C overnight. The slides were then visualized by the diaminobenzidine method.

For Oil Red O staining to examine triglyceride accumulation, livers were frozen in liquid nitrogen and embedded in OTC (optimum cutting temperature) compound. After staining, these sections were washed and embedded.

### Measurements of serum parameters and hepatic triglyceride, cholesterol and non-esterified fatty acids (NEFAs)

Serum insulin and ALT levels were assayed with the Ultra-sensitive mouse insulin Elisa kit (Morinaga Yokohama, Japan) and the Transaminase C-II Test Wako kit (Wako, Osaka, Japan), respectively. Triglycerides, cholesterol and NEFAs in the livers were measured with the triglyceride E-test (Wako Osaka, Japan), T-choE (Wako) and NEFA C test (Wako) kits, respectively.

### Quantitative real time reverse transcription PCR

Total RNA was extracted from mouse livers using Sepasol reagent (Nakalai Tesche, Kyoto, Japan). Template cDNA was obtained using total RNA employing a Verso cDNA synthesis kit (Thermo Scientific), and quantitative real time PCR was carried out using SYBR Green PCR master mix (Invitrogen, Tokyo, Japan). The designed primers were as follows; Collagen1a1 (F: GAGCGGAGAGTACTGGATCG R: GCTTCTTTTCCTTGGGGTTC), Collagen1a2 (F: CCGTGCTTCTCAGAACATCA R: GAGCAGCCATCGACTAGGAC), SMA (F: ACCAACTGGGACGACATGGAA R: TGTCAGCAGTGTCGGATGCTC), tissue inhibitor of metalloproteinase 1 (TIMP1) (F: ATTCAAGGCTGTGGGAATG R: CTCAGAGTACGCCAGGGAAC), monocyte chemotactic protein-1 (MCP-1) (F: AGGTCCCTGTCATGCTTCTG R: TCTGGACCCATTCCTTCTTG), interleukin-1β [interleukin (IL)-1 β] F: TGGGCCTCAAAGGAAAGAAT R: CTTGGGATCCACACTCTCCA), IL-6 (F: CCATCCAGTTGCCTTCTTGG R: TCCACGATTTCCCAGAGAACA), IL-12 (F: TGGAGCACTCCCCATTCCTA R: TGAGCTTGCACGCAGACATT), transforming growth factor (TGF) (F: GGAAGGACCTGGGTTGGAAG R: GGACAACTGCTCCACCTTGG), F4/80 (F: TCTGGGGAGCTTACGATGGA R: TAGGAATCCCGCAATGATGG) AND GAPDH (F: TGATGGGTGTGAACCACGAG R: GGGCCATCCACAGTCTTCTG).

### Statistical analysis

Results are expressed as mean ± SE. Statistical significance was assessed using ANOVA followed by the Tukey HSD test. Values of p < 0.05 were taken to indicate a statistically significant difference. Due to software limitations, we gave p < 0.0001 whenever the p value was smaller than 0.0001.

## Results

Mice were injected with both 120 mg/kg NA and 100 mg/kg STZ twice, 2 days apart (Fig. 1a). Half of these mice were treated with luseogliflozin (NA/STZ/HFDT/Luseo) and the other half was left untreated (NA/STZ/HFDT). While the blood glucose levels of the control mice, not given NA, STZ, or luseogliflozin, receiving only the ND, remained at approximately 150 mg/dL for the entire 8 weeks, NA/STZ/HFDT mice showed elevations to between 230 and 410 mg/dL (Fig. 1b). In contrast, the blood glucose level of the NA/STZ/HFDT/Luseo group was normalized to nearly that of the control group. The fasting serum insulin level of the NA/STZ/HFDT mice was approximately 40 % of that in control mice, and did not differ from that of NA/STZ/HFDT/Luseo mice (Fig. 1c). Interestingly, despite there being no significant differences in body weights among the three groups, liver weight and the serum ALT level were higher in the NA/STZ/HFDT than in the control mice, while showing normalization in the NA/STZ/HFDT/Luseo group (Fig. 1d, e). On the other hand, serum triglyceride and cholesterol levels were slightly higher in the NA/STZ/HFDT/Luseo than in the NA/STZ/HFDT group (Fig. 1f).

**Fig. 2:**
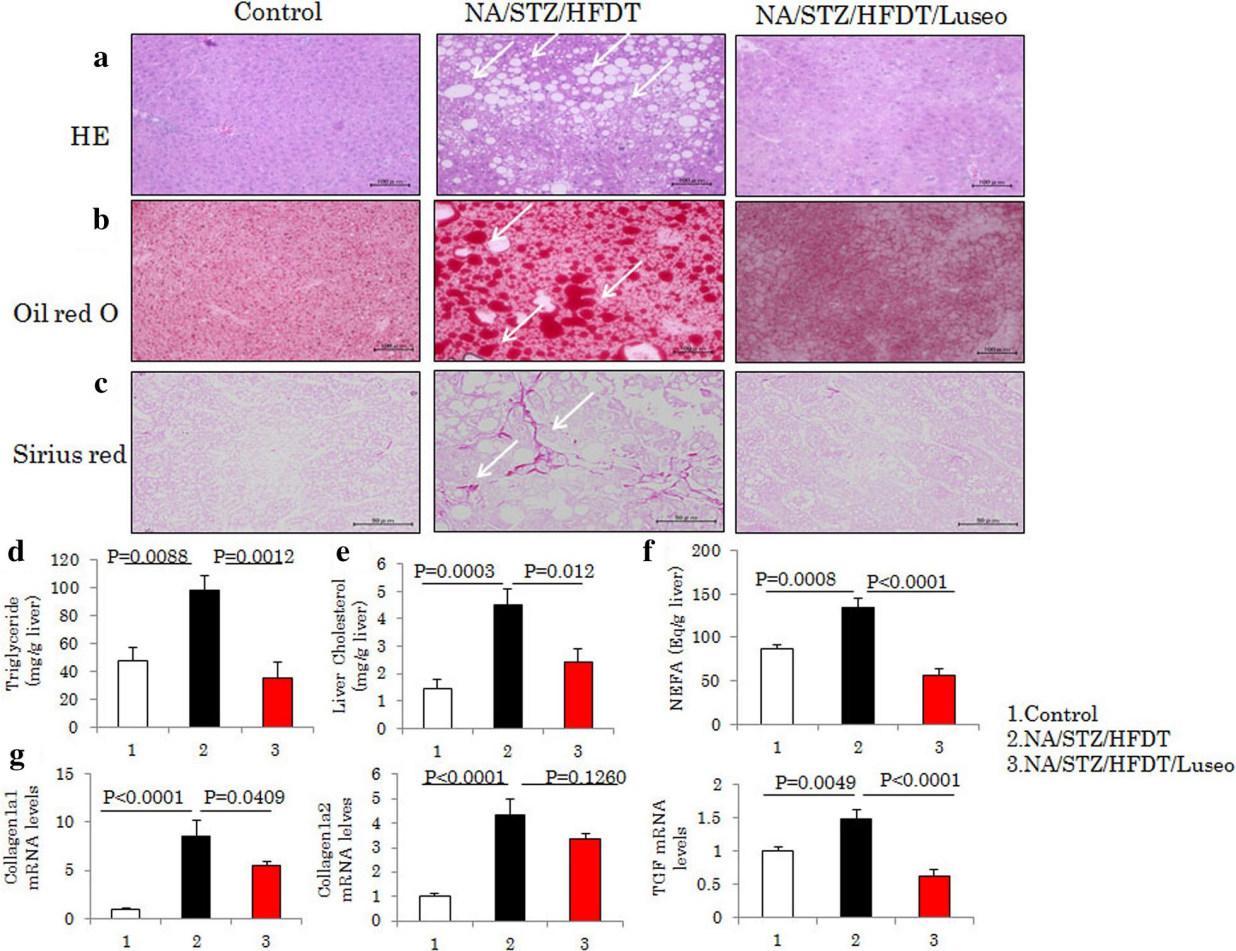
Luseogliflozin normalized HFDT feeding-induced hepatosteatosis and fibrosis. **a**–**c** Staining with HE, Oil red O and Sirius red, respectively. Representative photographs of each group are shown. **d–f** The amounts of triglyceride, cholesterol and NEFA in the livers. **g** mRNA levels of collagen 1, collagen 2 and TGF. All data are shown as means + SEM

Hematoxylin eosin staining of the livers from the control mice showed only modest steatosis. In contrast, the livers of NA/STZ/HFDT mice had developed marked macrovesicular steatosis, and the administration of luseogliflozin reduced this steatosis (Fig. 2a). Consistent with the HE staining results, Oil Red O staining revealed that the livers of control mice had only sparse lipid droplets, while NA/STZ/HFDT mice showed abundant lipid droplets. As expected, NA/STZ/HFDT/Luseo mice showed minimal lipid accumulation (Fig. 2b).

In contrast, the livers of NA/STZ/HFDT/Luseo mice showed much less hepatic accumulation of triglyceride, cholesterol and NEFA than those of the NA/STZ/HFDT mice not given luseogliflozin (Fig. 2d, e, f). Sirius red staining revealed marked liver fibrosis in the NA/STZ/HFDT mice, while there was almost no collagen deposition in the livers of NA/STZ/HFDT/Luseo mice (Fig. 2c). Consistent with the results of Sirius red staining, marked increases in mRNA expressions of collagen1a1/1a2 in the NA/STZ/HFDT mice as compared with the control mice, were partially but significantly normalized by luseogliflozin treatment (NA/STZ/HFDT/Luseo) (Fig. 2g). The TGF mRNA level was also elevated in the NA/STZ/HFDT mice, but showed normalization in the NA/STZ/HFDT/Luseo mice (Fig. 2g).

**Fig. 3:**
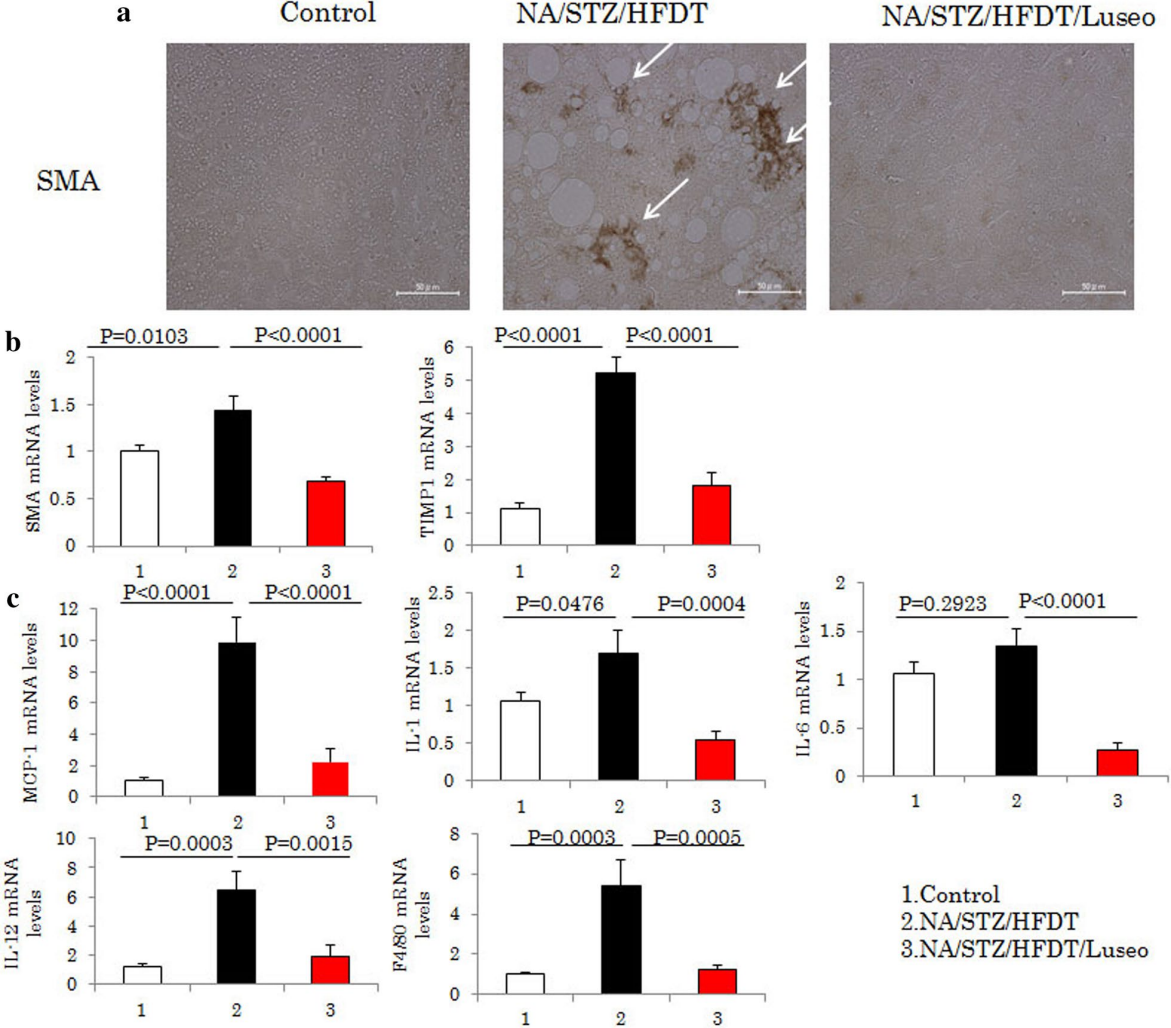
Luseogliflozin suppresses HFDT-induced activation of hepatic stellate cells and inflammation in the liver. **a** Immunostaining with anti-SMA antibody, as a marker of stellate cell activation. Representative photographs of each group are shown. **b** mRNA levels of SMA and TIMP1. **c** The mRNA levels of MCP-1, IL-1β, IL-6 IL-12 and F4/80. All data are shown as means + SEM

Since activation of hepatic stellate cells reportedly plays a critical role in the development of hepatic fibrosis [13, 14], immunofluorescence staining was carried out using anti-SMA antibody. Although the livers of control mice had few SMA-positive cells, numerous SMA positive cells were observed in the livers of HFDT-fed mice, and this finding was normalized by luseogliflozin administration (Fig. 3a). These immunochemical staining data were supported by the analysis of mRNA expressions of SMA and TIMP1, expressions of which were elevated in the NA/STZ/HFDT mice, but normalized in the NA/STZ/HFDT/Luseo mice (Fig. 3b). The expressions of cytokines such as MCP-1, IL-1β, IL-6, IL-12 and F4/80 were also markedly elevated in the NA/STZ/HFDT mice, but normalized in the NA/STZ/HFDT/Luseo mice (Fig. 3c). These results indicate that luseogliflozin treatment not only reduced lipid accumulation but also suppressed the inflammation in the livers of these mice.

## Discussion

In this study, we endeavored to prepare model mice suffering from both diabetes mellitus and NASH. As we previously found that blood glucose levels were raised only slightly by HFDT feeding, the mice in these experiments were treated with NA and STZ to moderately reduce insulin secretory capacity and thereby raise their blood glucose concentrations.

Our results using this diabetes plus NASH model showed clearly that luseogliflozin not only normalized elevated glucose concentrations, but also almost completely suppressed the development of NASH based on the histochemical findings, as well as measurements of hepatic lipid content and various serum parameters indicative of liver injury, fibrosis and inflammation. On the other hand, the NA/STZ/HFDT/Luseo group showed mild increases in serum TG and NEFA levels, as compared to the NA/STZ/HFDT group.

This phenomenon might be attributable to increased expression levels of scavenger receptor CD36 in the liver. Indeed, CD36 expression levels in the NA/STZ/HFDT group were approximately triple those in the control group, but were normalized by luseogliflozin treatment (data not shown).

Therefore, the livers of NA/STZ/HFDT mice may vigorously take up lipids from blood, resulting in decreased of blood TG and NEFA levels.

Interestingly, despite liver weights being significantly reduced in the NA/STZ/HFDT/Luseo as compared with NA/STZ/HFDT mice, the overall reduction in body weight was very slight and did not reach statistical significance. One possible explanation of the effect of luseogliflozin on NASH would be that excessive lipid accumulation in the NASH liver might be preferentially reversed, or even catabolized, in the energy deficient state induced by luseogliflozin administration. Another possible mechanism involves glucagon. Very recently, it was reported that SGLT2 is expressed in glucagon-secreting α cells of pancreatic islets and that SGLT2 inhibitors thus promote glucagon secretion [8]. An increased serum glucagon concentration may be the key to liver-dominant reduction of lipid accumulation.

Regarding the pathogenesis of NASH, the “two-hit theory” has been proposed as a mechanism underlying the development of this disorder. The first and second hits involve simple steatosis, while factors such as oxidative stress and inflammatory cytokines exacerbate NASH once it has developed [13, 15–18]. A previous study found that ipragliflozin had no significant effect on either inflammation or ALT and AST elevations in MCD diet-induced NASH model rats [5]. Their results are obviously different from ours showing luseogliflozin administration to exert a favorable effect on both the first and the second hit. The MCD diet-induced NASH model showing marked body weight loss is artificial, however, and does not reflect the pathogenesis of human NASH [19]. In addition, as the MCD diet fed mouse does not show glucose elevation or insulin resistance, it is not regarded as an appropriate model for investigating the effects of SGLT2 inhibitors. We consider beneficial SGLT2 inhibitor effects to not be limited to the amelioration of steatosis, with reductions in hepatic inflammation, cell death and fibrosis also being obtained, as indicated by our results.

Therefore, this is the first clear demonstration of the favorable effects of administering SGLT2 inhibitors, for the treatment of NASH accompanying diabetes mellitus, using a rodent model. We anticipate that a future clinical study will demonstrate the usefulness, for treating NASH, of SGLT2 inhibitor agents.

## Abbreviations

NAFLD: non-alcoholic fatty liver disease
NASH: non-alcoholic steatohepatitis
SGLT: sodium glucose cotransporter
STZ: streptozotocin
ND: normal chow diet
HFDT: high fat diet containing trans fatty acids
ALT: alanine aminotransferase
NA: nicotinamide
SMA: smooth muscle actin
NEFA: non-esterified fatty acids
IL: interleukin
TGF: tumor growth factor
TIMP: tissue inhibitor of metalloproteinases
MCP-1: monocyte chemoattractant protein 1
